# What drives parents’ use of air quality indexes during wildfire smoke events: predictors of index knowledge, frequent checking, and following health guidance

**DOI:** 10.1007/s11111-025-00491-w

**Published:** 2025-04-14

**Authors:** Catherine E. Slavik, Daniel A. Chapman, Stephanie E. Cleland, Ellen Peters

**Affiliations:** 1 Center for Science Communication Research, School of Journalism and Communication, University of Oregon, 1275 University of Oregon, Eugene, OR 97403 USA; 2 Faculty of Health Sciences, Simon Fraser University, Burnaby, Canada; 3 Legacy for Airway Health, Centre for Lung Health, Vancouver Coastal Health Research Institute, Vancouver, Canada; 4 Department of Psychology, University of Oregon, Eugene, USA

**Keywords:** Wildfire smoke, Environmental health, Air quality index, Smoke preparedness, Psychosocial factors

## Abstract

Wildfire smoke poses a serious and growing health threat to communities in the United States (US), Canada, and beyond. Some populations—including children—are especially susceptible. Air Quality Indexes (AQIs) can inform parents about local air quality during smoke events and offer guidance on actions that protect children. In July–August 2023, parents from Oregon, Washington, California, and British Columbia (BC) (*n* = 2100) participated in an online cross-sectional study. Binary and ordinal logistic regression models were used to examine geographic, demographic, and psychosocial predictors of three dependent variables: knowledge of where to check AQI information, frequent checking of AQI information during wildfire seasons, and adherence to AQI health messages around reducing/rescheduling outdoor physical activity. Smoke-exposure analysis indicated widespread potential exposures to wildfire smoke across all four jurisdictions. Nonetheless, parents in BC, on average, were less likely to report knowing where to check AQI information, checked less frequently, and were less likely to adhere to AQI guidance than parents in the three US states. Adherence to AQI health messages did not differ by jurisdiction in the presence of other covariates, suggesting parents are equally likely to follow AQI guidance when they know where to find it and check it. Other consistent predictors of the three dependent variables included experience with prior smoke-related health impacts, smoke risk perceptions, and use of internet/mobile applications as sources of smoke information. These findings indicate that increased promotion of AQIs may benefit parents in some regions during wildfires. Future evaluations of smoke education initiatives could help health agencies share effective practices across jurisdictions and target interventions to increase AQI adoption.

**Supplementary information:**

The online version contains supplementary material available at 10.1007/s11111-025-00491-w.

## Introduction

Wildfire activity has surged across western North America, significantly degrading air quality in communities throughout the United States (US) and Canada (Wilmot et al., 2022). The record-breaking Canadian wildfires of 2023 demonstrated that even populations previously considered safe from wildfire smoke events, such as those living along the Eastern Seaboard, will need to contend with the threat of wildfire smoke going forward (Yu et al., 2024). Climate change is one of the major drivers of these trends, making wildfire smoke events more intense and more frequent (Liu et al., 2021). In fact, health costs associated with wildfire smoke are projected to become among the most significant and costly consequences of climate change (Qiu et al., 2024).

While smoke from wildfires can impact everyone, certain populations are particularly sensitive. A growing body of literature has linked exposure to wildfire smoke to an increased risk of adverse health outcomes among susceptible groups such as children and adolescents (Zhang et al., 2024). Children’s lungs are still developing, they spend more time outdoors, and they breathe faster, inhaling more air relative to their size compared to adults, leading to higher doses of smoke (Hauptman et al., 2020; Slavik et al., 2023). Fortunately, various actions—such as limiting time outdoors during smoke events, using air purifiers, or donning respirators (i.e., N95 or P100)—can reduce children’s exposures to wildfire smoke (US Environmental Protection Agency and American Academy of Pediatrics’ Pediatric Environmental Health Specialty Units 2022).

However, the adoption of smoke-safe behaviors hinges on parents’ awareness of the presence of wildfire smoke in the air where they live. As a result, governments and health experts across North America often encourage parents to check their local Air Quality Index (AQI) to stay informed about levels of wildfire smoke in the air (Anderko et al., 2020; British Columbia Provincial Health Services Authority 2023). These indexes are available on websites and mobile applications (i.e., apps) and are often disseminated via local news sources (Coughlan et al., 2022). Though AQIs are used to communicate risk from multiple air pollutants with various sources, wildfire smoke contributes substantially to fine particulate matter (PM2.5) levels during wildfire seasons, driving air quality trends across the western US and Canada (British Columbia Centre for Disease Control, 2024; Burke et al., 2023). As a result, the AQI becomes a useful measure of PM2.5 levels attributable to wildfire smoke in the air during wildfire smoke events. Importantly, emerging evidence indicates that PM2.5 in wildfire smoke may be more harmful to health than equal doses of PM2.5 from other sources such as traffic-related air pollution (Aguilera et al., 2021).

AQIs are typically accompanied by health messages to guide decision-making given a certain value displayed. For example, when the US AQI—which ranges from 0 “Good” to 500 + “Hazardous”—reaches a Moderate risk level (between 51 and 100), the following guidance is issued: “If you are unusually sensitive to particle pollution, consider reducing your activity level or shorten the amount of time you are active outdoors” (AirNow 2024a). In Canada, the air quality *health* index (AQHI) is used similarly as a risk communication tool, though it is calculated differently and displays different values than the US AQI. During wildfire smoke seasons, Canada sometimes uses a modified version of the AQHI (the AQHI +), which adjusts the traditional AQHI scale to account for wildfire-specific PM2.5 levels in the air and reports the higher value between the AQHI or AQHI + (Ministry of Environment and Climate Change Strategy 2024). Importantly, engagement with this kind of air quality information—be it an AQI, AQHI or AQHI + —during wildfire smoke events appears to promote the adoption of smoke-safe behaviors (Hano et al., 2020; Slavik et al., 2024). Thus, this paper will refer to them both simply as AQIs.

Unfortunately, many adults in the US and Canada are not aware of AQIs (D’Antoni et al., 2017; McCarron et al., 2023). In a large-scale study of more than 12,000 US adults, Mirabelli et al. (2020) found that nearly half were not familiar with the AQI. This limited awareness and use of AQIs diminishes their potential effectiveness in motivating people to take actions that reduce harmful exposures. In fact, air quality alerts issued by government agencies during air pollution episodes have not always led to improved population health outcomes because individuals either do not see the alerts or do not follow them (Chen et al., 2018; Mirabelli et al., 2018; Santana et al., 2021). The situation may be particularly concerning for parents, as the failure to take protective measures can result in elevated smoke exposure among children, potentially exacerbating respiratory issues and leaving lasting consequences on their health (Holm et al., 2021). Thus, understanding parents’ use of AQIs and adherence to their health messaging during wildfire smoke events—and increasing use and adherence as appropriate—is a public health priority.

To our knowledge, no previous studies have examined predictors of self-reported AQI use and adherence specifically during wildfire smoke episodes, the focus of the present study. However, previous studies have found that a range of demographic and psychosocial factors influence AQI use and adherence to *general* air pollution health guidance outside of wildfire smoke events. For example, higher-income parents and community members tend to report more frequent use of AQIs (Radisic & Newbold, 2016). Additionally, people with pre-existing health conditions‒as well as parents of children with pre-existing health conditions‒appear to be more likely to check air quality information and adhere to health guidance around reducing exposures to air pollution (Delmas & Kohli, 2020; McCarron et al., 2024; Wen et al., 2009). Furthermore, the medium used to disseminate air quality information plays a role; receiving information from mobile apps or through advice from health professionals predicts greater adherence to AQI health messaging (D’Antoni et al., 2017; Keegan & Rahman, 2021; Riley et al., 2021).

Geographic differences in AQI use and adherence during smoke episodes are also less studied, despite the fact that air quality issues vary by location. Awareness of AQIs appears to be higher in areas with more significant air quality issues, likely because people’s proximity to pollution impacts their risk perceptions, which in turn motivates information-seeking as a threat-coping response (Barton Laws et al., 2015; Mirabelli et al., 2020; Yang et al., 2014). For example, individuals living in the western US—where wildfires are more common—have reported more frequent adoption of protective behaviors like staying indoors when air quality is poor in comparison to individuals living elsewhere in the US (Del Ponte et al., 2022). Air quality information-seeking also likely varies across different regions due to socio-cultural factors, such as differences in prior wildfire smoke experiences (Rose et al., 2017) and varying access to environmental health promotion programs by jurisdiction (Radisic et al., 2016). However, a significant gap remains in understanding how AQI use and adherence vary by location during wildfire smoke events and identifying the factors contributing to these geographic differences. While wildfire smoke disregards geographic boundaries, public health agencies must manage risks within their jurisdictions. If populations in some regions are less proactive in protecting themselves from wildfire smoke or are less aware of protective measures, targeted interventions in these areas may be needed.

This research investigates geographic, demographic, and psychosocial predictors of AQI knowledge, frequent AQI checking, and following AQI health guidance among parents residing in the US states of California, Oregon, and Washington and the Canadian province of British Columbia. The study findings provide new insights that can guide health agencies and researchers in designing and implementing targeted interventions to increase AQI use and adherence during wildfire smoke events, especially in regions that currently face a high health burden from wildfire smoke.

## Methods

### Study recruitment

A cross-sectional study was conducted online between July 2023 and August 2023 using a sample of *n* = 2100 parents/legal guardians. Eligibility criteria included completing a consent form, being aged 18 and over, and having at least one child aged 18 years or younger. Participants were recruited using two panel providers: Centiment for US-based participants and Leger Opinion for Canada-based participants. 1054 participants from Leger’s panel from British Columbia met the study’s eligibility criteria and clicked the link to the survey, of whom 500 provided complete high-quality responses (i.e., removing anyone who did not consent, did not pass 2 attention checks, and completed the study faster than one third of the median completion time). A total of 2941 participants from Centiment from California, Oregon, and Washington met the eligibility criteria and clicked the link to the survey, of whom 1600 provided complete high-quality responses using the same criteria. The four jurisdictions were selected for study since each has experienced significant wildfire smoke events in recent years.

We aimed to collect data balanced by state/province, gender, and income; thus, we established sampling quotas to try to achieve a roughly even split between residents of California, Oregon, Washington, and British Columbia, between men and women, as well as income earners above and below a household income of $80,000 CAD/USD. Although the median household incomes of parents of children aged below 18 in British Columbia, California, Oregon, and Washington range from approximately $92,000 to $108,900 CAD/USD (Statistics Canada, 2022; United States Census Bureau 2022), setting a lower income quota allowed us to capture data from more lower-income families who, on average, experience higher exposure to wildfire smoke (Krebs & Neidell, 2024; Vargo et al., 2023). Nonetheless, our sample may not fully represent the broader population of parents in these jurisdictions and since detailed population data specific to parents of children under 18 are not available, we were unable to construct sample weights.

Participants were approximately evenly distributed across the four jurisdictions: California (*n* = 539), Oregon (*n* = 524), Washington (*n* = 537), and British Columbia (*n* = 500). Figure A1 in the Online Appendix displays the geographic distribution of the participants across the four jurisdictions. Participation included a survey as well as an experiment (results published separately (Slavik et al., 2024). The median completion time was 13.6 min. Ethical approval was obtained from the University of Oregon (ID#00000870). All *N* = 2100 participants were retained in the dataset, regardless of whether missing data was present. In the full sample, there was no missing data on our dependent or independent measures. However, during the analysis process, some participants were excluded from analysis if they selected the “decline to respond” option for the income (*n* = 67) or gender identity (*n* = 3) questions. Additionally, for the analyses reported using the objective smoke exposure data, several participants were omitted for which we could not properly interpret their ZIP code response (*n* = 14). When performing analyses, only complete data for the relevant variables was analyzed. In the *Exposure Analysis* and *Survey Analysis* sections, we describe further how missing data were handled in our regression models and provide the final sample size for each analysis in the relevant results sections.

**Fig. 1:**
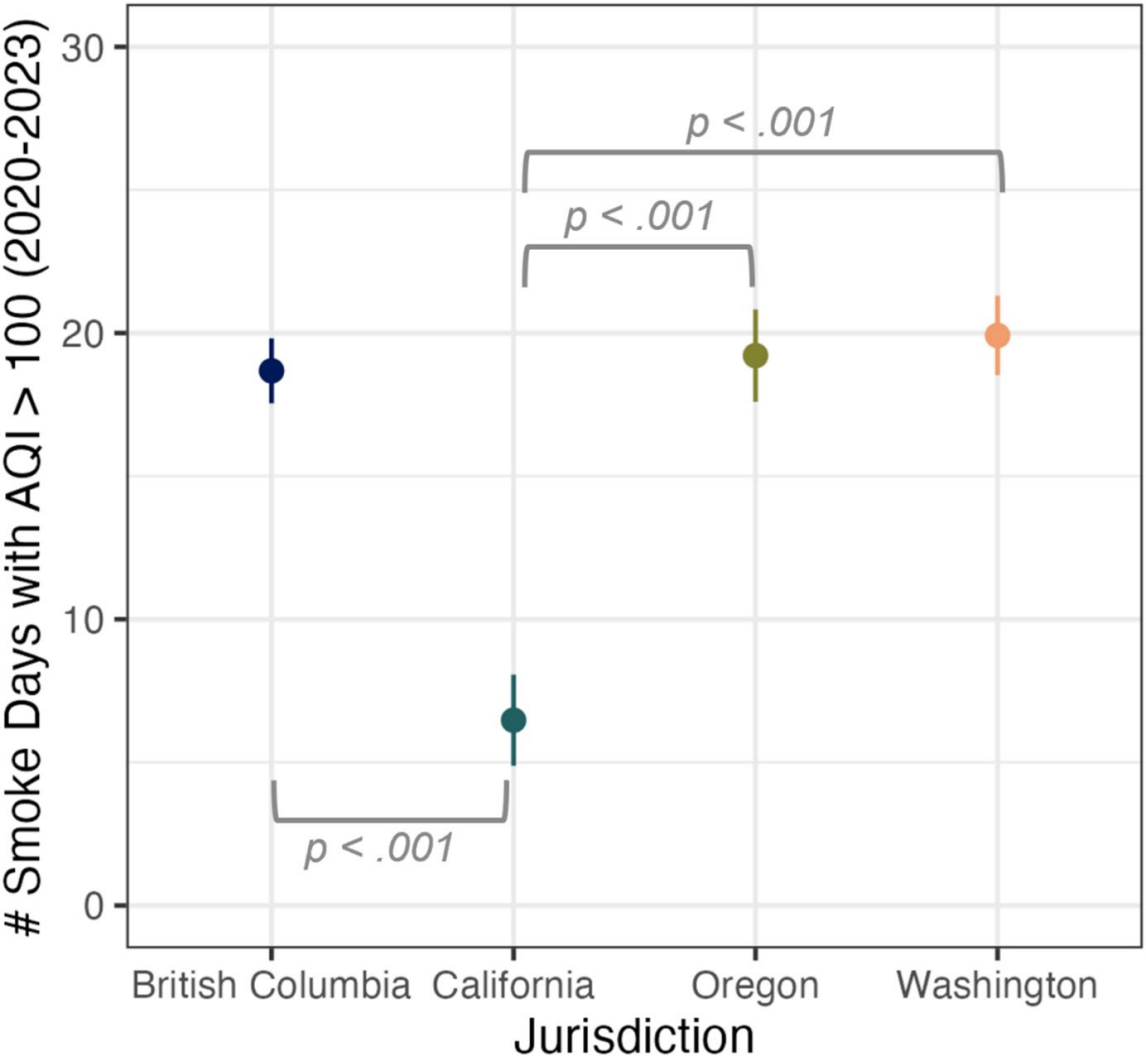
The average number of smoke days with US-AQI > 100 that occurred in each applicable postal code/ZIP code in our sample (*N* = 2086) between January 1 st 2020 until the day participants participated in the study in 2023. Values represent predicted means derived from a multilevel linear regression model, which controlled for average PM2.5 levels on non-smoke days and the distance to the nearest air monitoring station

### Variables

#### Dependent variables

This study had three dependent variables. The first was knowledge of where to get AQI information, which was asked with the following question: “Do you know where to check for daily air quality (health) index information?” The text in the parentheses of this question, and the two questions detailed below, was only seen by Canadian participants since Canada uses an AQHI rather than an AQI. Response options included yes, no, and unsure. The second dependent variable was participants’ self-reported frequency of checking air quality (health) index information during wildfire season with the following question: “During wildfire season, how often do you check the air quality (health) index?” Response options included never, once a month, once a week, daily, and unsure. The third variable measured whether participants followed AQI health guidance with the following question: “In general, do you follow air quality (health) index health messages which tell you when to consider reducing or rescheduling outdoor physical activity?” Response options included yes, no, and unsure.

#### Independent variables

The following demographic characteristics were measured and included as independent variables in our regression models: jurisdiction of residence, age, gender identity, education (bachelor’s degree or higher vs. lower), race (white vs. non-white), parent/child relevant health conditions, and prior smoke-related impacts on health. Participants could select multiple race/ethnicity categories; thus, if one of the categories selected was “white,” they were included in the “white” group. To assess health conditions that could be exacerbated by wildfire smoke, participants were asked to indicate whether they had any the following: asthma; chronic obstructive pulmonary disease; hypertension or high blood pressure; other heart-related disease, type II diabetes/metabolic syndrome/obesity; allergies related to the upper respiratory tract, eyes, and ears; and/or another chronic disease. Participants could select any number of conditions and were asked this question about both themselves as well as their child(ren). Previous health impacts from smoke exposure were measured using the item: “During previous wildfire seasons, did you or any members of your household ever experience levels of wildfire smoke in your community that you felt negatively influenced your health?” Response options included yes, no, and unsure.

The following psychosocial factors were also measured and included as independent variables in our regression models: risk perceptions about wildfire smoke and about general local air quality and health risk avoidance. Wildfire smoke risk perceptions were measured using the question: “If you or your family members had to spend the day outside, how much risk do you believe smoke from wildfires poses to you and your family’s health?” Response options included low risk, medium risk, elevated risk, high risk, very high risk, and extreme risk. General air quality risk perceptions were measured with the item: “In general, how do you rate the air quality in your neighborhood?” Response options included poor, fair, good, very good, excellent, and a unsure. Risk avoidance was measured using a Likert scale with the item: “If it concerns my health or the health of my family, I am someone who avoids taking risks.”

Frequency of receiving information about wildfire smoke from various sources was another psychosocial factor we assessed. Participants were asked how often they get information about wildfire smoke from different sources on a 4-point scale (1, never; 2, rarely; 3, often; 4, very often). The sources included the following: “my neighbors,” “my friends/family members,” “my employer,” “my doctor or other healthcare provider,” “internet websites (e.g., AirNow, purple air),” “applications (apps) on phone or tablet,” “local radio,” “local television,” “local newspapers,” “national radio,” “national television,” and “national newspapers.” From these measures, we created a series of composite indices by averaging together responses. Specifically, we grouped together “my neighbors,” “my friends/family members,” and “my employer” into an interpersonal sources measure. “Internet websites” and “applications (apps) on phone or tablet” were grouped together into a category for internet/apps. Local sources were grouped together (i.e., local radio, local television, and local newspapers) as were national sources (i.e., national radio, national television, and national newspapers). “My doctor or other healthcare provider” was analyzed on its own and not as part of a composite index.

### Exposure analysis

Given that AQI use and adherence is also likely influenced by the levels of wildfire smoke people experience, we assessed participants’ potential exposures to wildfire smoke by matching their postal codes and ZIP codes to daily data on smoke plumes from the National Oceanic and Atmospheric Administration’s Hazard Mapping System (HMS) Fire and Smoke Product (National Oceanic and Atmospheric Administration 2024) and PM2.5 concentrations from AirNow (AirNow 2024b) for 2020–2023. The HMS product uses satellite data to identify the shape and location of smoke plumes on any given day. AirNow provides data on daily average PM2.5 concentrations from air quality monitors run by agencies in the US and Canada. Because not every postal code/ZIP code has a local air quality monitor, we interpolated the daily PM2.5 concentrations to the postal code/ZIP code centroids using thin-plate spline regressions for each day using the *fields* package in R (Nychka et al., 2001). This approach ensured that each postal code/ZIP code had an estimate of average PM2.5 for any given day.

Data was available for 2086 participants after removing postal codes or ZIP codes that did not link to a defined geographic area or were not located in the four jurisdictions in the study. A given day in a postal code/ZIP code was identified as a “smoke day” if there was any smoke plume overhead on that day (i.e., light, medium, or heavy density smoke plume). Using the smoke plume and PM2.5 data, we identified the total number of smoke days each participant experienced between 2020 and 2023 (up until the day the participant started their survey) where daily concentrations of PM2.5 exceeded 35.5 μg/m^3^. To get a single value for each postal code/ZIP, we averaged the number of smoke days across all participants in each postal code/ZIP code. This approximately 3.5-year exposure period was selected to account for regional year-to-year variations in wildfire smoke that may have influenced participants’ AQI use prior to or at the time of survey administration. The 35.5 μg/m^3^ concentration was selected as it corresponds to a US-AQI value > 100 “Unhealthy for sensitive groups” risk level (and closely corresponds to the Canadian-AQHI +  > 3, a “Moderate” risk level) (AirNow 2024c; Taylor, 2018). On smoke days where the concentration of PM2.5 exceeds this threshold, air quality health guidance is issued that recommends for people to consider reducing or rescheduling outdoor activities (US EPA, 2023). While our measure included smoke plumes of any density, the vast majority of smoke days in our dataset with AQI > 100 (94.2%) either had a medium- or heavy-density smoke plume.

A multilevel linear regression model was fit to participant-specific data to examine jurisdiction differences in the number of smoke days with AQI > 100. Random intercepts were fit for each postal code/ZIP code. Two control variables were included in this smoke exposure model: the average PM2.5 concentration on non-smoke days between 2020 and 2023 (to adjust for non-smoke air pollution) and the distance of the postal code/ZIP code to the nearest air quality monitoring station. The latter was measured using data from AirNow Distance to the nearest monitoring station and was log-transformed to deal with substantial skew in the measure (log transforming was performed after first shifting the values by 1, because 0 values cannot be log-transformed). Adjusting for PM2.5 concentrations on non-smoke days was critical in this study to account for other non-wildfire smoke-related forms of air pollution, while controlling for distance to the nearest monitoring station was done to account for the fact people living in certain ZIP codes (e.g., rural ZIP codes in eastern Oregon) may be farther from the closest monitoring station than people living in other ZIP codes, such as urban areas. However, we also re-ran the model without the distance to monitoring station control to ensure this was not dramatically influencing the results. The results were substantively unchanged in terms of statistical significance of the pairwise comparisons among jurisdictions, and we therefore do not report on this further. Result plots, as well as maps of the average number of smoke days with AQI > 100, were generated in R using the *ggplot2* and *sf* packages (Wickham et al. n.d.).

### Survey analysis

To explore geographic, demographic, and psychosocial predictors of our three dependent variables (i.e., AQI knowledge, frequent AQI checking, and following AQI health guidance), we included data from the exposure analysis and from parents’ survey responses and included these in a series of models. First, we fit a logistic regression model to examine predictors of participants’ self-reported knowledge of where to get AQI information. Participants who answered with “unsure” were recoded as “no” for the analysis to facilitate the use of binary logistic regression. Table 2 provides the statistical summaries of the complete regression model. We initially ran a model with jurisdictions as the sole predictors (Model 1) and then added demographic factors into the model (Model 2), followed by the addition of psychosocial and control factors (Model 3). We took this three-stage approach to examine what impact these other predictors would have on the relationship between jurisdiction and our outcome measures. The three control variables included in Model 3 consisted of the total number of smoke days where the smoke concentrations exceeded 35.5 μg/m^3^ (i.e., the AQI was greater than 100), PM2.5 concentrations on non-smoke days, and distance to the nearest monitoring station. For each model, post-hoc pairwise comparisons were performed to compare knowledge across the four jurisdictions, and Tukey’s HSD *p*-value adjustment was applied to adjust for multiple post-hoc comparisons. Multicollinearity was assessed on Model 3 containing all predictors; the variance inflation factors were less than 5, suggesting minimal threat of multicollinearity issues.

When performing each model set (referred to in Tables 2, 3, and 4 as Models 1, 2, and 3), participants were dropped if they had missing data on any of the measures. This ensures, for example, that Models 1 and 3 were estimated based on the same total sample size. We report the total sample size in each Table’s caption. We followed this same missing data approach for each dependent measure (Tables 2, 3, and 4). Note that whereas Models 1 and 2 were logistic regressions, Model 3 is a multilevel logistic regression model. This third model is multilevel in nature because the control variables (days where AQI > 100, etc.) are clustered at the postal/ZIP code level, whereas the rest of the data are clustered at the individual level.

Next, we examined the effects of jurisdiction and our ensemble of other predictors on our second dependent variable, self-reported frequency of checking AQIs during wildfire season, among those who answered “yes” to the prior question about knowing where to check for AQI information. In this subset of participants, those who answered “unsure” to the frequency of checking air quality question were recoded as “never” for this analysis. The same three-stage modeling approach was adopted for this measure as was used for self-reported AQI knowledge. An ordinal logistic regression model was used to analyze the 4-point outcome scale for Models 1 and 2, and multilevel ordinal logistic regression model was used for Model 3. Table 3 provides the coefficients from the model. To aid in the interpretation of the coefficients for the location differences, we also generated the means for each jurisdiction based on the sample size used in the analysis.

We investigated participants’ self-reported adherence to AQI health guidance (i.e., the third dependent variable) also only among the participants who indicated knowing where to check for air quality information. We used a binary logistic regression model for Models 1 and 2 and a multilevel binary logistic regression for Model 3; coefficients from the model are summarized in Table 4. We again followed the same three-stage modeling process as described above. Participants who answered “unsure” to the question about whether they follow AQI health messages regarding reducing or rescheduling outdoor physical activity were re-coded as “no” responses.

To examine potential explanations for why differences in AQI use and adherence appeared between the four jurisdictions, we also performed the following exploratory analysis: we selected three predictors that were consistently significant across all the models detailed above and ran models with these predictors serving as dependent variables and the four jurisdictions serving as the predictors. This approach allowed us to assess whether these key factors varied systematically across jurisdictions, providing insight into potential differences in wildfire smoke experiences and adaptive responses that may contribute to the observed variations in AQI use and adherence. These models included a binary logistic regression model, an ordinal logistic regression model, and a linear regression model. As above, any participants who answered with an “unsure” were recoded as “no” to facilitate the use of binary logistic regression.

## Results

### Participant characteristics

Parents’ average age was 39.24 (*SD* = 8.92) with a range of 18 to 77 years old. Median family income in the previous year (i.e., 2022) corresponded to a bracket of $80–89,999 CAD/USD. When participants were asked to score how often they got information about wildfire smoke from five sources (on a 4-point scale from 1, never, to 4, very often), the averages for the sources were as follows: interpersonal sources (mean (M) = 2.25, standard error (SE) = 0.02), internet/apps (M = 2.89, SE = 0.02), doctor or other healthcare provider (M = 2.03, SE = 0.02), local sources (M = 2.23, SE = 0.02), and national sources (M = 1.98, SE = 0.02). Other participant characteristics are reported in Table 1. Below, we report analyses conducted by jurisdiction, first examining wildfire smoke exposures, followed by the survey results.

**Table 1:** Survey participant characteristics (*n* = 2100). Percentages may not add up to 100 due to rounding

Participant characteristics	*n*	%
Age		
18–29	281	13
30–39	831	40
40–49	713	34
50 +	275	13
Gender		
Men	875	42
Women	1196	57
Other	26	1
Did not report	3	< 1
Household income (CAD/USD)		
< $50,000	570	27
$50,000–69,999	936	45
$70,000–89,999	279	13
> $90,000	248	12
Did not report	67	3
Education		
Less than bachelor’s degree	1141	54
Bachelor’s degree or higher	959	46
Ethnicity		
White	1496	71
Non-white	604	29
Parents and/or child with health condition (self-reported)		
Yes	1243	59
No	857	41
Prior experience with negative health effects from wildfire smoke		
Yes	1332	63
No/unsure	768	37
Wildfire smoke risk perceptions		
Extreme	169	8
Very high	289	14
High	380	18
Elevated	462	22
Medium	393	19
Low	407	19
Perceptions of local air quality (in general)		
Poor	84	4
Fair	328	16
Good	731	35
Very good	684	33
Excellent	259	12
Unsure	14	< 1
Avoids taking risks to health		
Strongly agree	606	29
Agree	1023	49
Neither agree nor disagree	327	16
Disagree	98	5
Strongly disagree	46	2

### Wildfire smoke exposure by jurisdiction

Wildfire smoke exposure was assessed by jurisdiction (based on participant postal codes/ZIP codes) to compare exposures in the Canadian province of British Columbia and the three US states and contextualize trends in AQI use. Exposures were assessed from the start of 2020 until the date of survey administration in 2023. After controlling for average PM2.5 concentrations on non-smoke days, as well as distance to the nearest air quality monitoring station, we found that, between 2020 and 2023, participants in Washington lived in areas with an average of 19.92 smoke days where the AQI exceeded 100 (SE = 0.71, min = 6 days, max = 38 days). On days where the US-AQI > 100 (and the Canadian-AQHI +  > 3), air quality index health guidance is issued recommending that people consider reducing or rescheduling outdoor activities. The level of exposure in Washington was followed by Oregon (M = 19.21 days, SE = 0.82, min = 3 days, max = 76 days) and British Columbia (M = 18.68 days, SE = 0.58, min = 1 day, max = 45 days); California had many fewer days (M = 6.47 days, SE = 0.81, min = 1 day, max = 78 days) (Fig. 1). Post-hoc pairwise comparisons using the Tukey HSD *p*-value adjustment indicated that smoke exposure among participants in Washington, Oregon, and British Columbia were all significantly higher than California (*p*’s < 0.001). Washington did not differ from Oregon (*p* = 0.909) or British Columbia (*p* = 0.439). British Columbia did not differ from Oregon (*p* = 0.946).

**Fig. 2:**
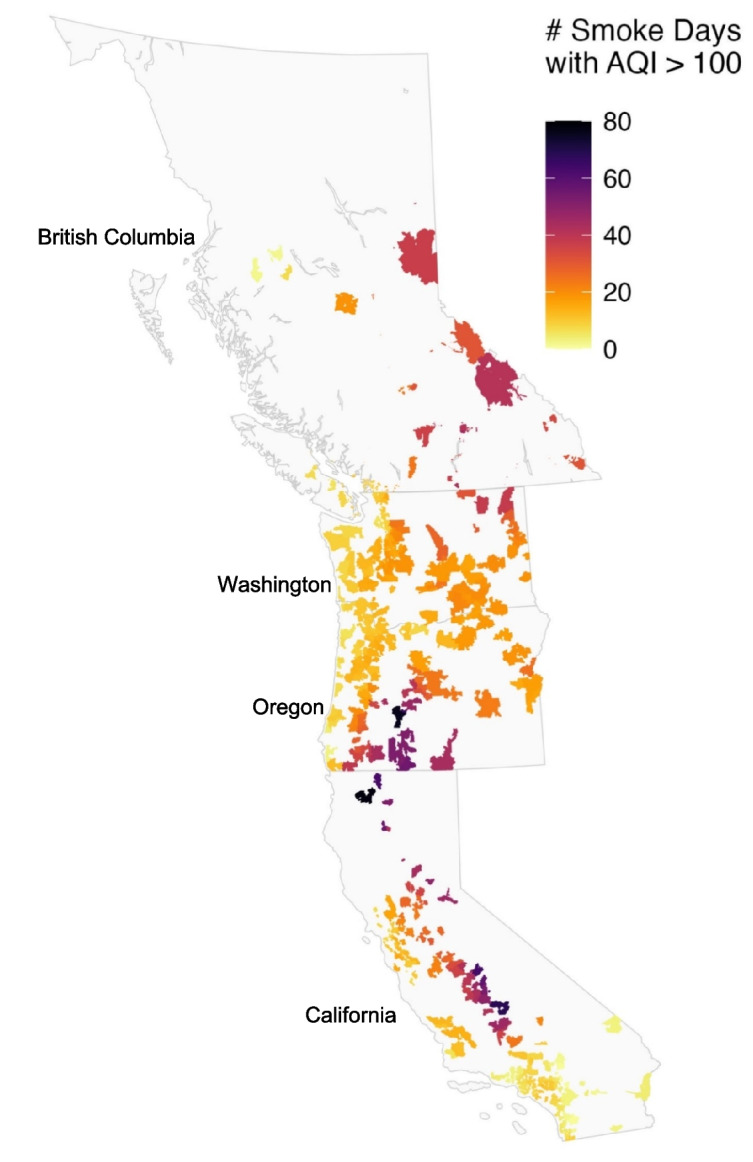
Map displaying the average number of wildfire smoke days (per postal code/ZIP code) where the US-AQI exceeded 100 (i.e., mean daily PM2.5 levels ≥ 35.5 ug/m^3^) between January 1 st 2020 until the day participants participated in the study in 2023

The number of smoke days with AQI > 100 varied considerably within the four jurisdictions between 2020 and 2023 (Fig. 2). All participants lived in a postal code/ZIP code area that experienced at least 1 smoke day with AQI > 100. Nearly half (46%) of participants experienced over 2 weeks of smoke days with AQI > 100 since 2020. Participants residing in a ZIP code located in northern California and central Oregon experienced the highest number of smoke days with AQI > 100; of the 36 participants that experienced over 50 smoke days where the AQI exceeded 100, 18 were located in California and 18 were in Oregon.

### Knowledge of where to get AQI information

Most participants in our sample reported knowing where to check for daily AQI information *(*76%, *n* = 1600), but some indicated not knowing (17%, *n* = 362) or were unsure (7%, *n* = 138) (data not shown)*.*

In Table 2, we present the results of our binary logistic regression predicting parents’ knowledge (vs. lack of knowledge) of where to get AQI information. Beginning with Model 1, which aimed to investigate the impact of parents’ jurisdiction on their knowledge of where to get AQI information, we found that compared to parents in British Columbia (reference group), parents in California, Oregon, and Washington were significantly more likely to report knowing where to check for AQI information (OR = 1.72, 95% CI [1.30, 2.28], OR = 1.79, 95% CI [1.35, 2.40], and OR = 1.96, 95% CI [1.47, 2.62], respectively).

**Table 2:** Binary logistic regression results predicting parents’ knowledge (vs. lack of knowledge) of where to get AQI information (*n* = 2044). Odds and 95% confidence intervals are reported

Parameter	Model 1 (Tjur’s *R*^2^ = 0.01)	Model 2 (Tjur’s *R*^2^ = 0.08)	Model 3 (Nakagawa’s conditional *R*^2^ = 0.38)
(Intercept)	2.12 [1.76, 2.58]	1.17 [0.64, 2.12]	0.01 [0.00, 0.05]
Jurisdiction			
California (vs. BC)	**1.72 [1.30, 2.28]**	**1.66 [1.22, 2.25]**	1.76 [0.97, 3.20]
Oregon (vs. BC)	**1.79 [1.35, 2.40]**	**1.74 [1.27, 2.38]**	**1.50 [1.03, 2.18]**
Washington (vs. BC)	**1.96 [1.47, 2.62]**	**1.96 [1.44, 2.66]**	**1.62 [1.13, 2.32]**
Demographic factors			
Age		0.99 [0.98, 1.00]	1.00 [0.99, 1.02]
Other gender (vs. man)		2.26 [0.71, 10.13]	1.88 [0.46, 7.67]
Woman (vs. man)		0.87 [0.69, 1.09]	0.79 [0.61, 1.04]
Bachelor’s degree (vs. less than bachelor’s degree)		**2.34 [1.86, 2.96]**	**1.95 [1.49, 2.57]**
White (vs. non-White)		**1.49 [1.17, 1.88]**	**1.49 [1.13, 1.95]**
Health condition (vs. not)		1.07 [0.85, 1.34]	0.98 [0.75, 1.26]
Prior smoke-related health impacts (vs. not)		**2.55 [2.04, 3.20]**	**1.90 [1.46, 2.48]**
Psychosocial factors			
Positive air quality perception			**1.25 [1.10, 1.43]**
Smoke risk perception			**1.12 [1.02, 1.22]**
Health risk avoidance			1.09 [0.95, 1.24]
Frequent use of interpersonal sources for smoke information			**0.75 [0.60, 0.95]**
Frequent use of internet/apps for smoke information			**3.48 [2.85, 4.24]**
Frequent use of primary care provider for smoke information			1.06 [0.90, 1.26]
Frequent use of local news sources for smoke information			1.09 [0.83, 1.37]
Frequent use of national news sources for smoke information			0.99 [0.77, 1.27]
Control factors			
Number of smoke days with AQI > 100 (*z*-score)			**1.21 [1.04, 1.40]**
Average PM2.5 on non-smoke days (*z*-score)			0.89 [0.70, 1.13]
Distance to nearest monitoring station (log + 1)			0.86 [0.75, 1.00]
Random effects			
Postal code intercept SD	NA	NA	0.45

For jurisdiction, the reference category is British Columbia (BC). For gender, the reference category is men. Predictors based on model parameters, with confidence intervals that do not include one, appear in bold text. Knowledge of where to get AQI information was analyzed using a binary yes/no outcome. Tjur’s *R*^2^ is an *R*^2^ implementation for binary logistic regression models. Nakagawa’s *R*^2^ is an implementation for mixed models. Conditional *R*^2^ takes both random and fixed effects into account

Reflecting these differences, parents in Washington had the highest probability of knowing where to check for daily AQI information with a predicted probability of 80.61% (SE = 1.72%), followed by Oregon (79.21%, SE = 1.81%), California (78.45%, SE = 1.79%), and British Columbia (67.98%, SE = 2.12%). Pairwise comparisons with Tukey’s HSD *p*-value adjustment indicated that the likelihood of parents in British Columbia knowing where to check AQI was significantly lower than that of parents in Washington (*p* < 0.001), Oregon (*p* < 0.001), and California (*p* = 0.001). Washington did not differ from Oregon (*p* = 0.944) nor California (*p* = 0.821). Oregon and California did not differ (*p* = 0.991).

In Model 2 (Table 2), we added demographic predictors to evaluate the added explanatory power of demographic characteristics in predicting parents’ knowledge of where to get AQI information. Post-hoc pairwise comparisons using the Tukey HSD *p*-value adjustment were found to be consistent with the results in Model 1. More specifically, the probability of knowing where to check for daily AQI information among parents in British Columbia was significantly lower than in Washington (*p* < 0.001), Oregon (*p* = 0.003), and California (*p* = 0.006). Washington did not differ from Oregon (*p* = 0.883) nor California (*p* = 0.742). Oregon and California also did not differ (*p* = 0.992).

Finally, in Model 3 (Table 2) where we included all jurisdiction, demographic, psychosocial, and control predictors into the model to explore their effects on parents’ knowledge, the Tukey HSD-adjusted post-hoc pairwise comparisons indicated that knowledge of where to get AQI information in British Columbia remained significantly lower than in Washington (*p* = 0.044). Differences in AQI knowledge between parents in British Columbia and those in Oregon and California were reduced to non-significance (*p* = 0.154 and *p* = 0.249, respectively). Washington again did not differ from Oregon (*p* = 0.980) nor California (*p* = 0.993), and Oregon did not differ from California (*p* = 0.947). Among the demographic factors included in the model, having a bachelor’s degree or greater, being white (vs. non-white), and having previous experience with smoke-related health impacts significantly predicted higher self-reported knowledge of where to get AQI information (Models 2–3, Table 2). The addition of psychosocial factors revealed that perceiving the air quality in your neighborhood as positive (outside of wildfire season) and reporting greater wildfire smoke risk perceptions were also associated with greater knowledge of where to get AQI information (Model 3, Table 2). Participants who reported frequently getting wildfire smoke information from interpersonal sources (e.g., family, friends) were less likely to know where to get AQI information, while those who reported frequently getting wildfire smoke information from the internet and mobile apps had much higher odds of knowing where to get AQI information (Model 3, Table 2). Number of smoke days where AQI > 100 had a small positive effect on the odds of self-reported knowledge of where to get AQI information.

### Frequency of checking AQI information during wildfire seasons

Out of the 1600 participants who knew where to check for AQI information, 61% (*n* = 971) said they checked daily, 25% (*n* = 399) said they checked once a week, 6% (*n* = 103) said they checked once a month, and 8% (*n* = 127) indicated they either never checked or were unsure how frequently they checked (data not shown). In Model 1 (Table 3), we again included parents’ jurisdiction as the sole predictor to explore its effects on parents’ frequency of checking AQI information during wildfire seasons. Compared to parents in British Columbia (reference group), parents in California, Oregon, and Washington were significantly more likely to report checking AQI information during wildfire seasons (OR = 1.85, 95% CI [1.40, 2.45], OR = 2.20, 95% CI [1.65, 2.94], and OR = 2.01, 95% CI [1.52, 2.66], respectively).

**Table 3:** Ordinal logistic regression results predicting frequency of checking AQI information during wildfire seasons *(n* = 1568)*.* Odds and 95% confidence intervals are reported

Parameter	Model 1 (Nagelkerke’s *R*^2^ = 0.03)	Model 2 (Nagelkerke’s *R*^2^ = 0.07)	Model 3 (Nakagawa’s conditional *R*^2^ = 0.24)
(Intercept 1 | 2)	0.14 [0.11, 0.18]	0.33 [0.18, 0.60]	19.27 [6.78, 54.82]
(Intercept 2 | 3)	0.28 [0.23, 0.35]	0.66 [0.37, 1.19]	41.26 [14.40, 118.27]
(Intercept 3 | 4)	1.11 [0.90, 1.36]	2.71 [1.51, 4.87]	200.52 [67.41, 596.44]
Jurisdiction			
California (vs. BC)	**1.85 [1.40, 2.45]**	**2.02 [1.50, 2.71]**	1.27 [0.77, 2.09]
Oregon (vs. BC)	**2.20 [1.65, 2.94]**	**2.09 [1.55, 2.83]**	**1.95 [1.39, 2.71]**
Washington (vs. BC)	**2.01 [1.52, 2.66]**	**2.01 [1.51, 2.69]**	**1.73 [1.27, 2.36]**
Demographic factors			
Age		1.00 [0.99, 1.01]	1.01 [1.00, 1.03]
Other gender (vs. man)		**4.89 [1.58, 21.51]**	**4.51 [1.23, 16.53]**
Woman (vs. man)		**1.28 [1.04, 1.59]**	1.17 [0.93, 1.48]
Bachelor’s degree (vs. less than bachelor’s degree)		**1.36 [1.11, 1.68]**	1.21 [0.63, 1.52]
White (vs. non-White)		0.97 [0.76, 1.22]	0.94 [0.73, 1.20]
Health condition (vs. not)		**1.37 [1.11, 1.69]**	1.15 [0.92, 1.44]
Prior smoke-related health impacts (vs. not)		**1.74 [1.40, 2.17]**	1.22 [0.97, 1.55]
Psychosocial factors			
Positive air quality perception			1.05 [0.94, 1.18]
Smoke risk perception			**1.29 [1.19, 1.40]**
Health risk avoidance			**1.24 [1.09, 1.40]**
Frequent use of interpersonal sources for smoke information			0.97 [0.79, 1.19]
Frequent use of internet/apps for smoke information			**2.34 [1.96, 2.79]**
Frequent use of primary care provider for smoke information			1.11 [0.96, 1.28]
Frequent use of local news sources for smoke information			**1.25 [1.01, 1.56]**
Frequent use of national news sources for smoke information			**0.77 [0.62, 0.94]**
Control factors			
Number of smoke days with AQI > 100 (*z*-score)			**1.14 [1.01, 1.29]**
Average PM2.5 on non-smoke days (*z*-score)			1.14 [0.94. 1.39]
Distance to nearest monitoring station (log + 1)			0.91 [0.80, 1.03]
Random effects			
Postal code intercept SD	NA	NA	0.11

For jurisdiction, the reference category is British Columbia (BC). For gender, the reference category is men. Predictors based on model parameters, with confidence intervals that do not include one, appear in bold text. The frequency of checking AQI information during wildfire seasons was scored on a 4-point scale (i.e., 1, never/unsure; 2, monthly; 3, weekly; 4, daily). Models only include those respondents who reported having knowledge of where to get AQI information. Nagelkerke’s *R*^2^ is an *R*^2^ implementation for ordinal logistic regression models. Nakagawa’s *R*^2^ is an implementation for mixed models. Conditional *R*^2^ takes both random and fixed effects into account

Pairwise comparisons using the Tukey HSD *p-*value adjustment revealed that parents in British Columbia reported taking this action significantly less often than parents in Washington (*p* < 0.001), Oregon (*p* < 0.001), and California (*p* < 0.001). The frequency of checking AQI information during wildfire seasons among parents in Washington did not differ from those in Oregon (*p* = 0.921) nor in California (*p* = 0.935), and parents’ checking from Oregon did not differ from California (*p* = 0.620). The means by jurisdiction were as follows for parents in Oregon (M = 3.48, SE = 0.04), Washington (M = 3.46, SE = 0.04), California (M = 3.42, SE = 0.04), and British Columbia (M = 3.14, SE = 0.06) (data not shown).

In Model 2, we again added demographic factors to the model to evaluate the added explanatory power of demographic characteristics in predicting AQI checking. We found that the pairwise comparisons were consistent with Model 1 (Table 3). Specifically, the frequency of checking AQI information during wildfire seasons among parents in British Columbia was significantly lower than parents in Washington (*p* < 0.001), Oregon (*p* < 0.001), and California (*p* < 0.001). Parents’ AQI checking in Washington did not differ from parents in Oregon (*p* = 0.993) nor in California (*p* = 1.00), and Oregon did not differ from California (*p* = 0.996).

In Model 3, we included all jurisdiction, demographic, psychosocial, and control predictors into the model to explore their effects on parents’ frequency of checking AQI information during wildfire seasons. The pairwise comparisons were consistent with the previous models (Table 3) in that parents from British Columbia checked the AQI less frequently than those in Washington (*p* = 0.003) and Oregon (*p* < 0.001). Differences in the frequency of checking the AQI between parents in British Columbia and those in California were reduced to non-significance (*p* = 0.782). Washington did not differ from Oregon (*p* = 0.877) nor California (*p* = 0.588), and Oregon did not differ from California (*p* = 0.291).

After demographic, psychosocial, and control factors were added to the model (Model 3, Table 3), results showed that having previous experience with smoke-related health impacts significantly predicted higher frequency of checking AQI information during wildfire seasons. As the coefficient for the difference between men and the “other” category for the gender variable was significant in Model 3 (Table 3), we also performed pairwise comparisons for gender using the Tukey HSD *p*-value adjustment. After the *p*-value adjustment, none of the gender differences was significant (*p* = 0.100). Therefore, we do not interpret gender differences for this measure any further.

Among parents who reported greater risk perceptions about wildfire smoke and higher health risk avoidance, each had significantly higher odds of frequently checking AQI information (Model 3, Table 3). Participants who reported frequently getting wildfire smoke information from the internet and mobile apps also had higher odds of frequently checking AQI information during wildfire seasons, as were parents who reported getting information from local news sources (Model 3, Table 3). Conversely, parents who reported getting information about wildfire smoke from national news sources had lower odds of frequently checking AQI information during wildfire seasons (Model 3, Table 3). The number of smoke days with AQI > 100 had a small positive effect on the odds of frequently checking AQI information.

### Adherence to AQI health messages

Of the 1600 participants who knew where to check for AQI information, 87% (*n* = 1386) reported following health messages that suggest when to reduce or reschedule outdoor physical activity, while 13% (*n* = 214) reported not adhering to these messages (or were unsure) (data not shown).

In Model 1 (Table 4), we included only jurisdiction as a predictor to examine its effect on the probability that parents reported adherence to AQI health messages recommending reduced or rescheduled outdoor physical activity. Compared to parents in British Columbia (reference group), parents in California, Oregon, and Washington were significantly more likely to report adherence to AQI health messages (OR = 2.12, 95% CI [1.39, 3.26], OR = 1.79, 95% CI [1.18, 2.71], and OR = 1.59, 95% CI [1.07, 2.37], respectively).

**Table 4:** Binary logistic regression results predicting adherence (vs. no adherence) to AQI health messages *(n* = 1568). Odds and 95% confidence intervals are reported

Parameter	Model 1 (Tjur *R*^2^ = 0.01)	Model 2 (Tjur *R*^2^ = 0.06)	Model 3 (Nakagawa’s conditional *R*^2^ = 0.40)
(Intercept)	4.31 [3.29, 5.73]	2.25 [0.95, 5.34]	0.01 [0.00, 0.05]
Jurisdiction			
California (vs. BC)	**2.12 [1.39, 3.26]**	**2.50 [1.59, 3.95]**	1.22 [0.53, 2.80]
Oregon (vs. BC)	**1.79 [1.18, 2.71]**	**1.62 [1.04, 2.53]**	1.45 [0.84, 2.51]
Washington (vs. BC)	**1.59 [1.07, 2.37]**	**1.54 [1.01, 2.34]**	1.30 [0.79, 2.13]
Demographic factors			
Age		0.98 [0.96, 1.00]	0.99 [0.97, 1.01]
Other gender (vs. man)		2.16 [0.57, 14.27]	1.85 [0.34, 10.11]
Woman (vs. man)		**1.80 [1.30, 2.48]**	**1.70 [1.16, 2.48]**
Bachelor’s degree (vs. less than bachelor’s degree)		**1.85 [1.34, 2.56]**	**1.46 [1.01, 2.13]**
White (vs. non-White)		1.07 [0.75, 1.51]	1.07 [0.71, 1.61]
Health condition (vs. not)		1.24 [0.90, 1.71]	0.93 [0.65, 1.35]
Prior smoke-related health impacts (vs. not)		**2.70 [1.96, 3.72]**	**2.12 [1.44, 3.13]**
Psychosocial factors			
Positive air quality perception			1.05 [0.87, 1.27]
Smoke risk perception			**1.24 [1.08, 1.42]**
Health risk avoidance			**1.79 [1.46, 2.18]**
Frequent use of interpersonal sources for smoke information			0.94 [0.68, 1.30]
Frequent use of internet/apps for smoke information			**1.61 [1.24, 2.08]**
Frequent use of primary care provider for smoke information			**1.71 [1.31, 2.22]**
Frequent use of local news sources for smoke information			1.40 [0.97, 2.01]
Frequent use of national news sources for smoke information			0.93 [0.65, 1.32]
Control factors			
Number of smoke days with AQI > 100 (*z*-score)			1.03 [0.84, 1.27]
Average PM2.5 on non-smoke days (*z*-score)			1.18 [0.83, 1.67]
Distance to nearest monitoring station (log + 1)			0.97 [0.79. 1.19]
Random effects			
Postal code intercept SD	NA	NA	0.67

For jurisdiction, the reference category is British Columbia (BC). For gender, the reference category is men. Predictors based on model parameters, with confidence intervals that do not include one, appear in bold text. Adherence to AQI health messages was analyzed using a binary yes/no outcome. Models only include those respondents who reported having knowledge of where to get AQI information. Tjur’s *R*^2^ is an *R*^2^ implementation for binary logistic regression models. Nakagawa’s *R*^2^ is an implementation for mixed models. Conditional *R*^2^ takes both random and fixed effects into account

Reflecting these differences, parents in California had the highest probability of reporting adherence to AQI health messages with a predicted probability of 90.12% (SE = 1.46%), followed by Oregon (88.50%, SE = 1.60%), Washington (87.26%, SE = 1.62%), and British Columbia (81.16%, SE = 2.16%). After applying the Tukey HSD *p*-value adjustment to Model 1’s coefficients, parents’ reported adherence to AQI health messages in British Columbia was significantly lower than Oregon (*p* = 0.030) and California (*p* = 0.003), but not lower than Washington (*p* = 0.100). Washington did not differ from Oregon (*p* = 0.949) nor California (*p* = 0.561), and California and Oregon did not differ (*p* = 0.877) (Table 4).

In Model 2, we added demographic factors to the model to explore their effects on adherence to AQI health messages. We found that the probability of reporting adherence to AQI health messages among parents in British Columbia significantly differed from California (*p* < 0.001), but not Oregon (*p* = 0.146) or Washington (*p* = 0.183) after applying the Tukey HSD *p*-value adjustment. Washington did not differ from Oregon (*p* = 0.996) or California (*p* = 0.158), and California and Oregon did not differ (*p* = 0.289) (Table 4).

In Model 3, we included all jurisdiction, demographic, psychosocial, and control predictors into the model to explore their effects on the probability that parents reported adherence to AQI health messages. After applying the Tukey HSD *p*-value adjustment, we found that the probability of reporting adherence to AQI health messages among parents in British Columbia did not differ from parents in California (*p* = 0.966), Oregon (*p* = 0.531), or Washington (*p* = 0.735) (Table 4). Washington did not differ from Oregon (*p* = 0.972) or California (*p* = 0.999), and California and Oregon did not differ (*p* = 0.974) (Table 4).

Among the demographic predictors examined, parents with a Bachelor’s degree or higher were more likely to report adhering to AQI health messages, as were women, and parents who reported previous wildfire smoke health impacts (Model 2, Table 4). When psychosocial factors were added to the model, higher wildfire smoke risk perceptions and health risk avoidance also significantly predicted adherence to AQI health messages (Model 3, Table 4). As the gender coefficient for men vs. women was significant in Model 3, we also performed post-hoc pairwise comparisons among the gender groupings, again applying the Tukey HSD *p*-value adjustment. The predicted probabilities were as follows: other (94.44%, SE = 4.31%), woman (92.33%, SE = 1.03%), man (87.39%, SE = 1.60%). The difference between men and women was significant after applying the *p*-value correction (*p* = 0.006). Men did not differ from the “other” category (*p* = 0.525), nor did women differ from the “other” category (*p* = 0.908). Parents who reported receiving information about wildfire smoke from either internet/mobile apps or from their primary care provider had higher odds of reporting adherence to AQI health messages (Model 3, Table 4). None of the control factors had a significant effect on message adherence.

### Key predictors of AQI use and adherence stratified by jurisdiction

In an attempt to identify possible reasons for differences in AQI use and adherence between the jurisdictions, exploratory analyses were carried out to examine differences across three key variables: previous smoke-related health impacts, smoke risk perceptions, and frequent use of internet/mobile apps as a source of wildfire smoke information. These three variables were consistent predictors of the three dependent variables (i.e., AQI knowledge, frequent checking, following health messaging) based on model outputs (Tables 1, 2, 3). By comparing how key variables like parents’ smoke risk perceptions vary across jurisdictions, we aimed to investigate possible underlying geographic factors that might contribute to differences in AQI use and adherence.

**Fig. 3:**
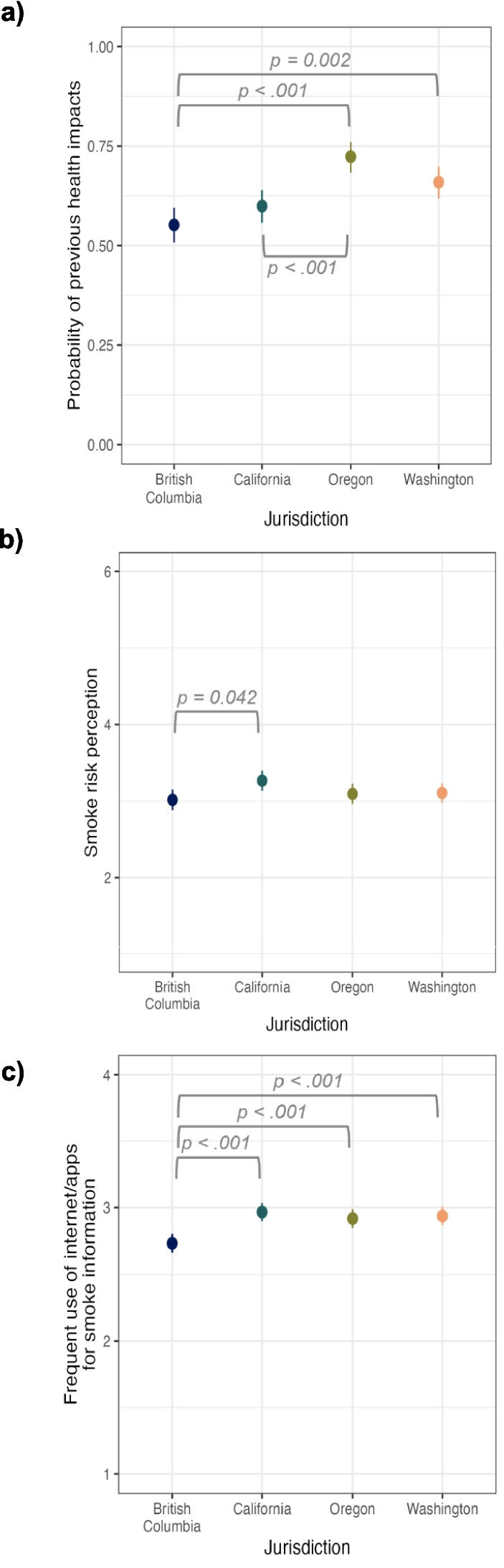
Mean participant responses by jurisdiction for (**a**) previous smoke-related health impacts, (**b**) smoke risk perceptions, and (**c**) frequent use of internet/mobile apps as a source of wildfire smoke information

Parents in British Columbia were the least likely to report previous health impacts from smoke (55.20%, SE = 2.22%), followed by California (59.93%, SE = 2.11%), Washington (65.92%, SE = 2.05%), and Oregon (72.33%, SE = 1.95%) (Fig. 3a). Post-hoc pairwise comparisons using the Tukey HSD *p*-value adjustment indicated that Oregon was higher than California (*p* < 0.001) and British Columbia, (*p* < 0.001), but not Washington (*p* = 0.109). Washington was higher than British Columbia (*p* = 0.002), but not California (*p* = 0.175). British Columbia did not differ from California (*p* = 0.414).

Parents in British Columbia had the lowest smoke risk perception score on a 6-point scale (M = 3.02, SE = 0.07), followed by Oregon (M = 3.09, SE = 0.07), Washington (M = 3.11, SE = 0.07), and California (M = 3.27, SE = 0.07) (Fig. 3b). Post-hoc pairwise comparisons using the Tukey HSD p-value adjustment indicated that California significantly differed from British Columbia, *p* = 0.042. British Columbia did not differ from Oregon (p = 0.900) nor Washington (*p* = 0.794). Oregon did not differ from Washington (*p* = 0.996) nor California (*p* = 0.209). Washington did not differ from California (*p* = 0.305).

Parents in British Columbia reported least frequent use of internet/apps for wildfire smoke information on a 4-point scale (M = 2.73, SE = 0.04), followed by parents in Oregon (M = 2.92, SE = 0.04), Washington (M = 2.94, SE = 0.04), and California (M = 2.97, SE = 0.03) (Fig. 3c). Post-hoc pairwise comparisons using Tukey’s HSD *p*-value adjustment indicated that the parents’ reported frequency of using internet/apps to obtain smoke information was significantly lower in British Columbia than Washington (*p* < 0.001), Oregon (*p* = 0.001), or California (*p* < 0.001). Oregon did not differ from Washington (*p* = 0.978) nor California (*p* = 0.754). Washington and California did not differ (*p* = 0.934).

## Discussion

Protecting children from wildfire smoke requires parents and caretakers to adopt certain actions that reduce exposure, such as reducing time outdoors or using portable air purifiers (Holm et al., 2021). As expected, our wildfire smoke exposure analysis revealed significant exposures across the four jurisdictions. Thus, it is crucial to ensure that parents in these areas know how to check air quality levels and follow public health guidance based on current smoke conditions. This study aimed to investigate geographic, demographic, and psychosocial factors influencing parents’ knowledge of where to check AQI information, frequent checking of AQI information during wildfire seasons, and adherence to AQI health messages around reducing or rescheduling outdoor physical activity. Across all four jurisdictions, parents who reported having previous experiences with smoke-related health impacts (among themselves or their family members), higher wildfire smoke risk perceptions, or frequently got wildfire smoke information from online sources like the internet/mobile apps were also more likely to know where to check the AQI, frequently check it, and follow AQI health guidance.

This research also examined regional differences in key predictors of AQI use and adherence to explore why parents in some jurisdictions may be more likely to use AQIs and adhere to their messaging during wildfire smoke events. From a public health standpoint, increasing engagement with AQIs could motivate more parents to respond to unhealthy levels of wildfire smoke by adopting behaviors that mitigate children’s exposures to smoke, thereby reducing risks to health (Slavik et al., 2023). This study’s results revealed that parents in the US states of California, Oregon, and Washington, on average, scored higher on AQI use and adherence measures than parents in British Columbia, Canada. After including various demographic, psychosocial, and wildfire smoke exposure covariates in our models, results indicated that parents in British Columbia were significantly less likely to report knowing where to check the AQI than parents from Washington. They also checked the AQI less frequently during wildfire smoke seasons compared to parents in Oregon and Washington. However, differences in AQI health messaging adherence were no longer significantly different between parents in British Columbia and parents in the three US states after applying the full host of predictors. The absence of significant differences in AQI adherence suggests that parents are equally likely to follow AQI health guidance across jurisdictions when they know where to find the AQI and check it. This result would indicate that differences in AQI knowledge and checking could be due to potential differences in information dissemination practices across jurisdictions, rather than actual behavioral differences in protecting against wildfire smoke exposure. These findings are encouraging as they suggest that making AQI tools and resources more visible and accessible in regions where citizens currently do not use them could increase AQI adoption during wildfire smoke seasons.

Our exploratory analyses demonstrated that parents across all four jurisdictions considered wildfire smoke to be similarly risky. Given that parents in British Columbia lived in areas that experienced a similar (or higher) number of smoky days in the three years preceding this cross-sectional study, one might expect AQI use and adherence to be more similar between the parents we surveyed in Canada and the US. However, parents in British Columbia were less likely to report having experienced previous health impacts from smoke. In the Appendix, we report regional differences for other demographic and psychosocial characteristics, which revealed that parents in British Columbia also reported a lower rate of health conditions compared to parents in the three US states. Therefore, it is possible that parents in British Columbia (or their child/children) who were, on average, healthier, have less motivation to use or adhere to AQIs. If true, this finding would present a significant opportunity to enhance public education on wildfire smoke since smoke exposure has been linked to health effects like declines in lung function and respiratory ER visits even among healthy children without conditions like asthma (da Jacobson et al., 2012; Leibel et al., 2020). Still, unlike some previous research (Delmas & Kohli, 2020; Wen et al., 2009), we did not find a consistent association between AQI use and/or AQI adherence and having a relevant health condition, suggesting individuals’ perceived *experiences* with health impacts were a stronger motivator for AQI adoption than the mere presence of a health condition that could be exacerbated by exposure to wildfire smoke.

Interestingly, other studies have found that people’s perceived severity of unhealthy air quality—including believing that symptoms were caused by air pollution—predicts AQI use and adherence and other smoke-safe behaviors (Berlin Rubin & Wong-Parodi, 2022; D’Antoni et al., 2017). Thus, it is also possible that parents in British Columbia were less likely to report having personally experienced previous health impacts from smoke and less likely to use or adhere to AQIs, because of differences in awareness surrounding the severity and scope of health impacts linked to wildfire smoke exposure. In other words, while parents in British Columbia may recognize that wildfire smoke poses a health risk in a general sense, they may not attribute the full range of potential health effects to smoke exposure, which could result in underreporting previous health impacts from smoke. Although the present study did not measure knowledge about wildfire smoke, future research should examine the impact of knowledge about smoke and health risks associated with exposure on AQI use and adherence.

Regional differences in the frequency of participants’ use of internet/mobile apps as a source of wildfire smoke information could also help to explain the differences in AQI use we observed between parents in British Columbia and the US states. Prior research has suggested that showing targeted, highly localized air quality information to users can increase engagement and adoption of protective actions (Keegan & Rahman, 2021; Riley et al., 2021). Information users also prefer sources like apps for their near real-time updates on wildfire smoke conditions (Prince et al., 2024; Walsh et al., 2022). Thus, more frequent use of online websites and/or apps displaying local smoke levels among parents in the three US states, relative to parents in British Columbia, may have contributed to their higher reported use of AQIs.

Our study has important implications for health agencies and experts investigating strategies to increase AQI adoption during wildfire smoke events. Findings suggest that in addition to demographic and psychosocial factors, geography may play a key role in expanding the use and impact of AQIs as regional differences appear to shape how people engage with air quality information. Specifically, the results indicate that increased promotion of Canada’s AQHI—and citizens’ awareness about where to check for it—may be needed in British Columbia. Based on our findings, online tools like existing websites and mobile apps could be used to increase AQHI engagement, at least among groups like parents who are more likely to use smartphones to seek health-related information online than other groups (e.g., seniors) (Jia et al., 2021). However, online air quality and smoke-specific information tools should be tested to ensure they are effectively promoting behavior change (Slavik et al., 2024).

Other jurisdictions, including the three US states we studied, may also need to expand wildfire smoke education initiatives and better target certain segments of the population. Notably, our study found that parents with lower education were less likely to know where to check the AQI and to follow health guidance. Moreover, we ran exploratory analyses on a subset of our sample of parents who did not know where to check for the AQI and who reported a health condition either among themselves or their child(ren), which could be exacerbated by wildfire smoke, who could benefit the most from targeted interventions going forward. We found that this sub-group of parents were on average, lower income (*p* < 0.001) and less educated (*p* < 0.001). This finding is especially worrisome given that previous research has shown that communities with lower socioeconomic status tend to experience higher exposures to wildfire smoke and higher risk of smoke-related diseases (Rappold et al., 2017). Thus, effectively communicating smoke conditions and increasing the adoption of smoke-safe behaviors in these areas, and among disadvantaged groups that may lack awareness of the AQI, is crucial to addressing smoke-related health inequities. Public health agencies and practitioners should continue to evaluate their public education programs on wildfire smoke, carefully examining AQI adoption in vulnerable communities, and share effective practices across jurisdictions.

This study has some important limitations to consider. The correlational nature of this study means that the relationships between AQI-related behaviors and geographic, demographic, and psychosocial factors are likely more complex than our models suggest. Moreover, in multiple regression analyses, the estimated effect of a given variable is assessed while holding other variables constant (i.e., at their mean values). For example, when examining the effect of frequently using one source of wildfire smoke information (e.g., internet/apps), the frequency of use of other sources is held at its average level. Given the correlational design, caution is warranted in interpreting the directionality of these relationships. Future research should continue to explore how these relationships operate to provide health agencies with a clearer understanding of the factors that motivate AQI adoption.

Furthermore, the findings from this research may not be broadly applicable due to the sample consisting solely of parents living in wildfire-prone regions rather than a more representative sample of adults. AQI use and adherence may have been lower had we sampled a more general adult population, assuming that people without children might feel less urgency in monitoring air quality. For example, compared to results from Mirabelli et al. (2020), which suggested that approximately half of Americans were familiar with the AQI, our study found that approximately 76% of parents reported knowing where to check for daily AQI information. This higher proportion could be the result of participants in our study having a heightened awareness around air quality issues due to the increased frequency and severity of wildfires on the west coast of the US and Canada. Future research should explore how other populations engage with air quality information, especially in regions where wildfire smoke events are less frequent.

There are also limitations associated with using self-reported data as it can be subject to certain biases such as social desirability (e.g., over-reporting use of AQI) or inaccurate recall of information (e.g., how often one checks wildfire smoke information). These biases could affect the reliability and validity of the study results; thus, the conclusions drawn from this research may not fully reflect the true behaviors of parents with respect to AQI use and adherence. Finally, our wildfire smoke exposure estimates were based on participants’ postal codes/ZIP codes, which may not accurately assess each participant’s true exposure—particularly if a participant recently moved to that location or spends considerable time elsewhere. However, measuring more precise individual exposures to wildfire smoke would not have been feasible for this study due to the extensive measurement and monitoring equipment required to track PM2.5 concentrations at the individual-level.

## Conclusion

This study explored differences in AQI use and adherence among parents in British Columbia, Canada, and three US states, California, Oregon and Washington—four jurisdictions that experience significant wildfire smoke seasons that are projected to worsen with climate change. On average, parents in British Columbia were less likely to know where to check AQI information, checked AQI information less frequently during wildfire seasons, and were less likely to report following AQI health messages regarding reducing or rescheduling outdoor physical activities, although differences in adherence between parents in British Columbia and US-based parents were non-significant after including other covariates in the model. Experiencing prior smoke-related health impacts from wildfires, having higher smoke risk perceptions, and frequent use of internet/mobile applications to obtain smoke information were also associated with higher AQI knowledge, frequent checking, and following AQI health messages. This research highlighted jurisdictional differences in AQI adoption during wildfire smoke events, which may help inform smoke-protective behaviors among certain groups of parents more effectively than others. Our findings suggested that implementing and encouraging the use of online tech-based interventions, as well as increasing awareness of wildfire smoke health impacts, could help increase the use of and adherence to AQIs. This study underscores the need for jurisdictions to evaluate their wildfire smoke education initiatives to increase the public’s knowledge of AQIs and promote the adoption of smoke-safe behaviors.

## Supplementary information

Below is the link to the electronic supplementary material.

ESM 1 (DOCX 207 KB)

## Data Availability

Data and study materials will be publicly available for download upon study publication at https://osf.io/jxkpg/files/osfstorage.
