# Supplementary material for: What drives parents’ use of air quality indexes during wildfire smoke events: predictors of index knowledge, frequent checking, and following health guidance
Source: Popul Environ. 2025 Apr 14;47(2):20. doi: 10.1007/s11111-025-00491-w (PMC11996998; doi:10.1007/s11111-025-00491-w)
Supplement: Supplementary file 1 — (DOCX 207 KB) [file 11111_2025_491_MOESM1_ESM.docx]

**Online Appendix**

**Figure 3.** Mean participant responses by jurisdiction for a) previous smoke-related health impacts, b) smoke risk perceptions, and c) reliance on internet/mobile apps to obtain wildfire smoke information.

A series of regression models were fit to examine differences in demographic factors (e.g., age, family income, education, etc.) as well as psychosocial factors (e.g., risk perception) between the jurisdictions represented in the study. The results of each model are described below.

A logistic regression model was fit to examine differences across jurisdictions in whether the parent or their child/children have relevant health conditions or not. Oregon had the highest rate (68.32%, SE = 2.03%), followed by Washington (63.12%, SE = 2.08%), California (56.03%, SE = 2.14%), and British Columbia (48.80%, SE = 2.24%). Post-hoc pairwise comparisons using the Tukey HSD p-value adjustment indicate that Oregon significantly differed from California and British Columbia (p’s < 0.001), but not Washington, *p* = 0.283. Washington different from British Columbia only, p < 0.001. British Columbia differed from Washington and Oregon (p’s < 0.001), but not California, p = 0.091. California differed from Oregon only, p < 0.001.

A linear model was fit to examine age differences across the jurisdictions. There were age differences such that British Columbia (*M* = 41.19, SE = 0.40) was slightly older than Washington (M = 39, SE = 0.38), California (*M =* 38.79, SE = 0.38), and Oregon (M = 38.10, SE = 0.39). Post-hoc pairwise comparisons using the Tukey HSD p-value adjustment indicate that British Columbia significantly differed from the three U.S. states (*p’s <* 0.001), but the three U.S. states did not differ from one another (*p’s* > 0.30).

An ordinal logistic regression model was fit to examine family income bracket differences across the jurisdictions. California had the highest average income (Mean = 9.81, SE = 0.17), followed by British Columbia (M = 9.11, SE = 0.16), Washington (M = 7.80, SE = 0.18) and Oregon (M = 6.88, SE = 0.17). Post-hoc pairwise comparisons using the Tukey HSD p-value adjustment indicate that all pairwise differences are significant at the *p <* 0.01 level.

A logistic regression model was fit to examine differences across jurisdictions in whether participants had obtained a bachelor's degree or greater or not. California had the highest probability of having a bachelor's degree (57.51%, SE = 2.13%), followed by British Columbia (56%, SE = 2.22%), Washington (40.22%, SE = 2.12%), and Oregon (29.20%, SE = 1.97%). Post-hoc pairwise comparisons using the Tukey HSD p-value adjustment indicate that all comparisons were significant at the p < 0.01 level except for British Columbia and California, which did not differ, *p* = 0.961.

Three separate logistic regression models were fit to compare the three gender identity categories. Specifically, one model compared males and females, one model compared males against the “other” category, and one compared females against the “other” category. In the male/female model, post-hoc pairwise comparisons indicated that California had fewer females (36.02%, SE = 2.08%) compared with Oregon (67.77%, SE = 2.07%), British Columbia (65.11%, SE = 2.15%), and Washington (63.04%, SE = 2.09%), with each difference significant at the p < 0.001 level. British Columbia, Oregon, and Washington did not significantly differ from one another, p’s > 0.30. Comparing males to the “other” category, Oregon (6.25%, SE = 1.82%) had a higher percentage of “other” responses than California (1.45%, SE = 0.64%), p = 0.029. None of the other pairwise comparisons were significant, p’s > 0.10. Comparing females to the “other” category, none of the post-hoc pairwise comparisons were significant, p’s > 0.20.

A logistic regression model was fit to compare whether participants identified as white or non-white. Oregon had the highest percentage of white participants (82.82%, SE = 1.65%), followed by Washington (77.47%, SE = 1.80%), British Columbia (63.20%, SE = 2.16%), and California (61.22%, SE = 2.10%). Post-hoc pairwise comparisons using the Tukey HSD p-value adjustment indicate that Oregon differed from the other three jurisdictions, *p’s <* 0.001. Washington differed from British Columbia and California (p’s < 0.001), but not Oregon, p = 0.129. British Columbia and California did not differ, p = 0.914.

An ordinal logistic regression model was fit to compare the risk aversion measure across jurisdictions. California (M = 4.01, SE = 0.04), Oregon (M = 3.97, SE = 0.04), Washington (M = 3.96, SE = 0.04) and British Columbia (M = 3.96, SE = 0.04) all had similar risk aversion scores, with none of the post-hoc pairwise comparisons being significant, p’s > 0.70.

An ordinal logistic regression model was fit to compare participants’ general air quality ratings in their area across jurisdictions. *N* = 14 participants were omitted from this analysis because they selected “unsure” for the measure. Washington had the highest air quality ratings (M = 3.43, SE = 0.04), followed by British Columbia (M = 3.40, SE = 0.05), Oregon (M = 3.38, SE = 0.04), and California (M = 3.15, SE = 0.05). Post-hoc pairwise comparisons using the Tukey HSD p-value adjustment indicate that California was significantly lower than British Columbia, Oregon, and Washington, p’s < 0.01. The other jurisdictional differences were not significant, p’s > 0.80.

A binary logistic regression model was fit to compare the probability of reporting previous health impacts due to smoke across the jurisdictions. For this analysis, anyone selecting “don’t know” (*n* = 131) were recoded as “no” responses. Oregon had the highest probability of reporting previous health impacts (72.33%, SE = 1.95%), followed by Washington (65.92%, SE = 2.05%), California (59.93%, SE = 2.11%), and British Columbia (55.20%, SE = 2.22%). Post-hoc pairwise comparisons using the Tukey HSD p-value adjustment indicate that Oregon was higher than California and British Columbia, *p’s* < 0.001, but not Washington, *p* = 0.109. Washington was higher than British Columbia, p = 0.002, but not California, p = 0.175. British Columbia did not differ from California, p = 0.414.

A linear regression model was fit to compare the frequency with which participants reported receiving information from interpersonal sources (neighbors, family and friends, employers) across the jurisdictions. California (M = 2.41, SE = 0.03) was highest on this measure, followed by Oregon (M = 2.27, SE = 0.03), Washington (M = 2.21, SE = 0.03), and British Columbia (M = 2.10, SE = 0.03). Post-hoc pairwise comparisons using the Tukey HSD p-value adjustment indicate that California was significantly higher than the other three jurisdictions, p < 0.05. Oregon was higher than British Columbia, p = 0.001, but did not differ from Washington, p = 0.509. Washington did not differ from British Columbia, p = 0.056.

An ordinal logistic regression model was fit to compare the frequency with which participants reported receiving information from their doctor across the jurisdictions. An ordinal logistic regression model was fit instead of a linear model because this is a single ordinal scale item, unlike the other information source composite measures. California was highest on this measure (M = 2.28, SE = 0.04), followed by Washington (M = 2.03, SE = 0.04), Oregon (M = 2.02, SE = 0.04), and British Columbia (M = 1.78, SE = 0.04). All pairwise comparisons using the Tukey HSD p-value adjustment were significant at the p < 0.001 level, except for Oregon and Washington which did not differ, p = 1.00.

A linear regression model was fit to compare the frequency with which participants reported receiving information from the local sources (local radio, television, and newspapers) across the jurisdictions. California was highest (M = 2.48, SE = 0.03), followed by Washington (M = 2.16, SE = 0.03), British Columbia (M = 2.13, SE = 0.04), and Oregon (M = 2.11, SE = 0.03). California significantly differed from each of the other three jurisdictions, p’s < 0.001. None of the other pairwise comparisons using the Tukey HSD p-value adjustment were significant, p’s > 0.70.

A linear regression model was fit to compare the frequency with which participants reported receiving information from the national sources (national radio, television, and newspapers) across the jurisdictions. California was again highest (M = 2.31, SE = 0.04), followed by Washington (M = 1.90, SE = 0.04), British Columbia (M = 1.85, SE = 0.04), and Oregon (M = 1.83, SE = 0.04). California significantly differed from each of the other three jurisdictions, p’s < 0.001. None of the other pairwise comparisons using the Tukey HSD p-value adjustment were significant, p’s > 0.40.


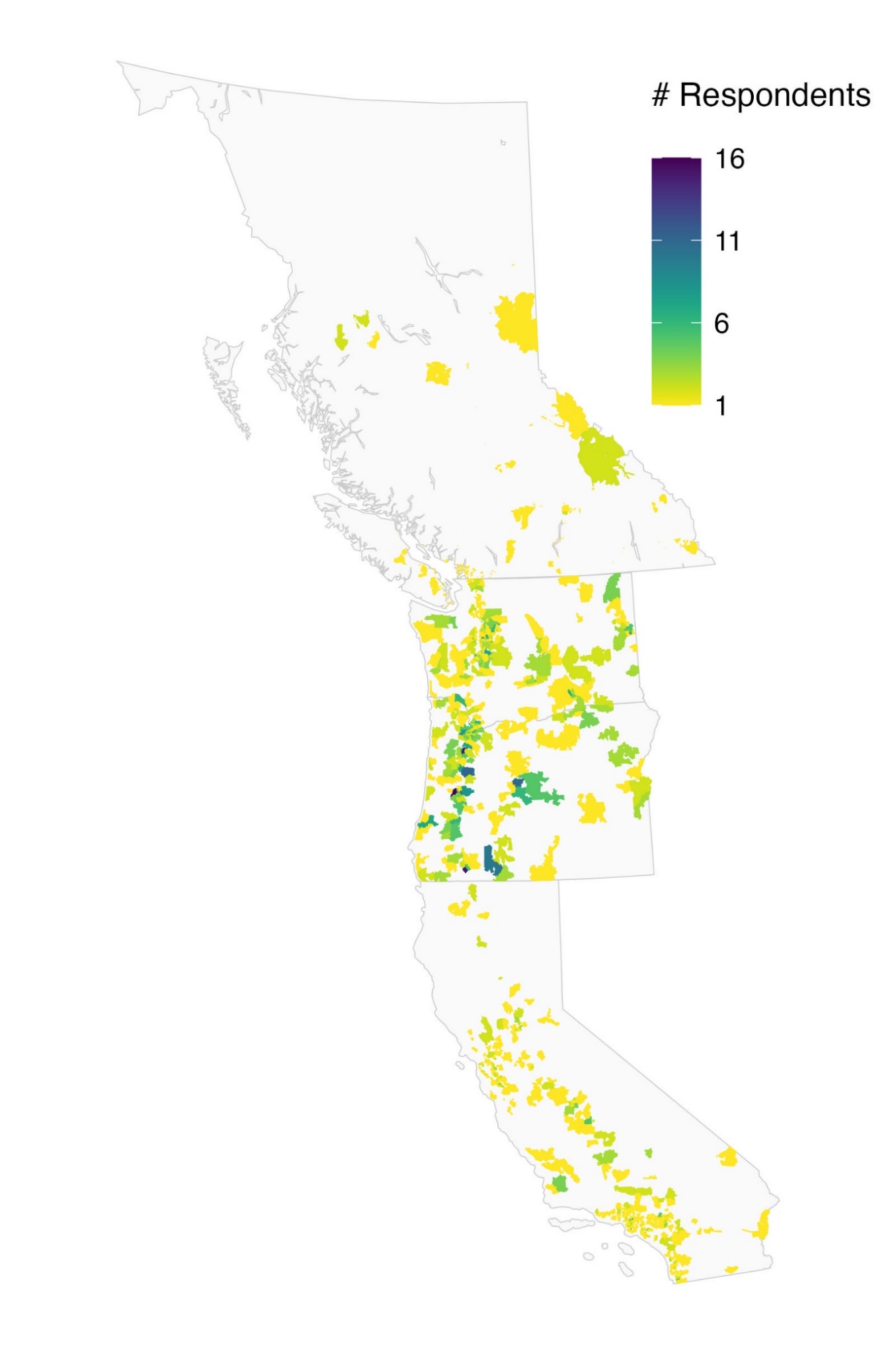


British Columbia

Oregon

California

Washington

**Figure A1.** Number of survey respondents per postal code/ZIP code area.
